# Interactions Between Non‐Native Western Mosquitofish and Native Bluegill Sunfish: Mesocosm Experiments

**DOI:** 10.1002/ece3.70508

**Published:** 2024-10-30

**Authors:** Jessica E. Rettig, Elizabeth P. Tristano, Anthony C. Burger, Geoffrey R. Smith

**Affiliations:** ^1^ Department of Biology Denison University Granville Ohio USA

**Keywords:** biotic resistance, competition, *Gambusia affinis*, intraguild predation, invasive species, *Lepomis macrochirus*

## Abstract

Aquatic ecosystems are often negatively affected by invasive species. However, biotic resistance by native species, either by competition or predation, can reduce the impacts of invasions by non‐native species. The Western Mosquitofish (*Gambusia affinis*) is one of the most impactful invasive species of freshwater fish and cause declines in native fish populations. Using two mesocosm experiments conducted in different years, we examined the ecological interactions between juveniles of the native fish, Bluegill Sunfish (*Lepomis macrochirus*), and adults of the invasive fish, *G. affinis*. We found evidence for interactions between *L. macrochirus* and *G. affinis*. However, interactions did not appear symmetric, with *L. macrochirus* generally more affected by intraspecific interactions than interspecific interactions whereas *G. affinis* was more affected by interspecific interactions than intraspecific interactions. The presence of either species of fish led to a decrease in the number of large zooplankton and a tendency for a decrease in the total number of zooplankton. Based on these results, native *L. macrochirus* appear to be able to reduce the ability of non‐native *G. affinis* to establish or maintain populations through both competition and predation (i.e., acting as an intraguild predator). The consistency of our results across both experiments, with their different designs and their occurring in different years, gives weight to these conclusions. The reduction of or prevention of establishment of populations of invasive *G. affinis* would likely benefit the aquatic communities of ponds with fish, especially small‐bodied native fish.

## Graphical abstract

Native Bluegill Sunfish had important competitive impacts on invasive Western Mosquitofish and also had intraguild predation effects that prevented successful recruitment of Western Mosquitofish. In contrast, Bluegill Sunfish were affected by intraspecific effects more than by interspecific effects from Western Mosquitofish. Our results suggest that native Bluegill Sunfish might provide a degree of biotic resistance to invasion by Western Mosquitofish in small temperate ponds.

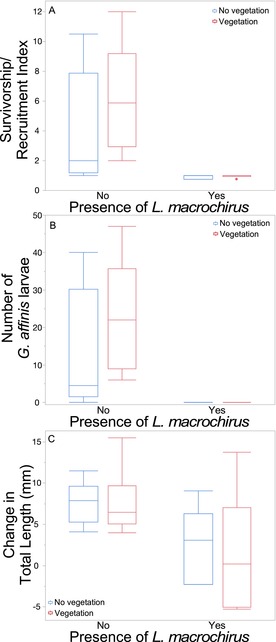

## 1 Introduction

Invasive species are a significant and widespread threat to aquatic ecosystems (Cambray 2003; Havel et al. 2015; Gangloff, Edgar, and Wilson 2016). The introduction of non‐native species, especially fish, into freshwater ecosystems can have unpredictable and extensive impacts on the ecosystem and native species (Cambray 2003). Predation and competition are the two most important impacts of non‐native fish on native ecosystems (Van der Veer and Nentwig 2015), and more observations on the interactions between native and non‐native fishes, especially their competitive interactions, are needed (García‐Berthou 2007; Almeida and Grossman 2012).

Western Mosquitofish (*Gambusia affinis*) and Eastern Mosquitofish (*G. holbrookii*) are two of the most invasive species of freshwater fish (Copp et al. 2009). These two species of mosquitofish are hardy and aggressive, adapt easily to a variety of environments, and reach high population densities rapidly, making them effective invaders (Pyke 2008; Rehage et al. 2020). The invasion of *Gambusia* spp. appears to have led to the extirpation, population declines, of native fish, or shifts in the distributions of native fishes (Goren and Galil 2005; Habit et al. 2010; Schumann et al. 2016; Ennen et al. 2021). The negative effects of mosquitofish on native fishes can arise due to competition, aggressive interactions, or predation (e.g., Mills, Rader, and Belk 2004; Goldsworthy and Bettoli 2006; Sutton, Zeiber, and Fisher 2013; Wedderburn et al. 2020). In particular, the consumption of the eggs or young of other fishes by *G. affinis* appears to often explain their negative impacts on native fish populations and communities (e.g., Rogowski and Stockwell 2006; Ayala et al. 2007; Laha and Mattingly 2007; Schumann, Hoback, and Koupal 2015). Indeed, the presence of *G. affinis* reduced the ability of some native fishes to produce juveniles or successfully reproduce (Sutton, Zeiber, and Fisher 2009; Goodchild and Stockwell 2016). For example, the survivorship of juvenile *Iotichthys phlegethontis* was reduced by a third in the presence of *G. affinis*, and at high *G. affinis* density, young‐of‐year *Iotichthys phlegethontis* had 100% mortality (Mills, Rader, and Belk 2004).

Biotic resistance can lead to failed invasions by non‐native species (Zenni and Nuñez 2013). In particular, native predators can limit the ability of invasive species to invade (e.g., de Rivera et al. 2005; Verhelst et al. 2016; Flaherty and Lawton 2019). Indeed, native fish can reduce the ability of invasive species to invade communities through predation (e.g., Britton 2012; Hill 2016; see also Deacon, Fraser, and Farrell 2023; Gu et al. 2023) and biotic resistance to invasive species in freshwater habitats appears to be stronger from predation rather than competition (Alofs and Jackson 2014). Native intraguild predators may be especially effective at resisting invasive species (e.g., Tuckett et al. 2021; Deacon, Fraser, and Farrell 2023). For example, intraguild predation by native species that are large enough to consume mosquitofish may limit their successful invasion and allow co‐existence (e.g., Henkanaththegedara and Stockwell 2013, 2014). Native competitor fishes can also resist the invasion of mosquitofish. For example, native *Galaxias maculatus* may be able to resist invasive *G. affinis* in clear water conditions due to their better foraging efficiency relative to *G. affinis* relative to turbid water (Abrahams, Bassett, and Montgomery 2017). Interference or physical harassment of non‐native fish by native fish can also injure and reduce the growth of the invader (Schofield et al. 2021).

Within their native range, *G. affinis* often co‐occurs with *Lepomis* spp., including Bluegill Sunfish (*L. macrochirus*) and Longear Sunfish (*L. megalotis*; e.g., Gelwick et al. 2001; Parham 2009; Matthews and Marsh‐Matthews 2011; Fisher, Kelso, and Rutherford 2012). In addition, *L. macrochirus* and other *Lepomis* spp. can use the same ponds and waterbodies as *G. affinis* in areas where both taxa are introduced (Moyle and Nichols 1973; Lynch 1988). The co‐occurrence of *Lepomis* spp. and *G. affinis* can persist within their native ranges (Hargrave and Taylor 2010) and in the non‐native range of *G. affinis* (Lynch 1988; Burskey and Simon 2008).

Native *Lepomis* species, including *L. macrochirus*, might affect the ability of *G. affinis* to establish or maintain populations. Both fish species have been observed to exploit similar food resources, including zooplankton and aquatic macroinvertebrates (Hurlbert and Mulla 1981; Lazzaro et al. 1992; Rettig 2003; Rettig et al. 2023), and both species are known to have significant effects on zooplankton communities (Nowlin and Drenner 2000; Fryxell et al. 2015; Geyer, Smith, and Rettig 2016; Rettig and Smith 2021). This dietary overlap may result in competition between *L. macrochirus* and mosquitofish, especially juvenile *L. macrochirus*. Previous studies have demonstrated this potential interaction between *Lepomis* sp. and *Gambusia* sp. For example, Green Sunfish (*Lepomis cyanellus*) and *G. affinis* compete, with both species negatively affected by the presence of the other, and their presence reduces the abundance and richness of invertebrates (Blaustein 1991). The presence of *L. cyanellus* also causes a habitat shift in *G. affinis* (Blaustein 1991). The presence of juvenile *L. macrochirus* reduced the foraging efficiency of male *G. affinis*, potentially reducing resource use, but not of females, and also reduced the aggressive interactions of *G. affinis* (Clemmer and Rettig 2019). However, in their native ranges, dollar sunfish (*Lepomis marginatus*) had no effect on *G. holbrooki* (Schofield et al. 2014).

The presence of vegetation can sometimes mediate the interactions among fishes. Vegetation can reduce predation risk for prey fish (e.g., Werner and Hall 1988; Santos et al. 2009; Alexander et al. 2015), the trophic structure of fish communities (Carey et al. 2010), or the growth of the fish (e.g., Crowder and Cooper 1982; Carey et al. 2010). However, interactions of *G.a ffinis* with other fish, including *L. cyanellus*, are sometimes not affected by vegetation or habitat complexity (Simkins and Belk 2017). *Lepomis macrochirus* are associated with submerged vegetation in some lakes (Werner, Hall, and Werner 1978; Kraus and Jones 2012), especially juveniles (Collingsworth and Kohler 2010). *Gambusia* spp. can also be found in submerged vegetation (Kraus and Jones 2012). Thus, the presence of vegetation or habitat structure might influence the interactions between *L. macrochirus* and *G. affinis*.

Using two mesocosm experiments conducted in two different years, we examined the effects of juvenile *L. macrochirus* and adult *G. affinis* on the growth and survival of each other. Our first experiment was conducted in the presence of artificial vegetation and the resource base for the fishes was limited to zooplankton. In our second experiment, we manipulated the presence and absence of artificial vegetation to examine the potential influence of vegetation on interactions between these two species. We also used more realistic vegetation in this second experiment. In addition, we allowed colonization of the mesocosm by insects and amphibians throughout the second experiment to more closely mimic the resource base for the fishes that would be found in natural ponds. The differences between the experiments thus allow us to potentially generalize our results more than either experiment on its own.

Because mosquitofish are known to be aggressive competitors and have been observed to reduce growth and cause high mortality in juvenile populations of other fish species (Laha and Mattingly 2007; Mills, Rader, and Belk 2004), we predicted that *L. macrochirus* would experience reduced growth in the presence of mosquitofish due to competition over a shared zooplankton resource (and insects and amphibians in Experiment 2). Also, because juvenile *L. macrochirus* prefer vegetated habitat with sufficient cover to avoid other fish (Casterlin and Reynolds 1978), we predicted that *L. macrochirus* that were exposed to both *G. affinis* and vegetation in Experiment 2 would experience a smaller decline in growth than *L. macrochirus* in the presence of *G. affinis* only. In addition, given the potential for resource competition and aggressive interactions between *G. affinis* and juvenile *L. macrochirus*, we predicted that *G. affinis* would show lower individual growth rates and survivorship in the presence of *L. macrochirus* compared to intraspecific competition.

## 2 Materials and Methods

We conducted our two mesocosm experiments in two different years at the Experimental Pond Array facility at the Denison University Biological Reserve, Granville, Licking County, Ohio. Below we describe the establishment of these two unique but similar experiments.

### 2.1 Experiment 1 (2010)

We filled 42 mesocosms (1136 L capacity, 63.5 cm height, 175 cm diameter) with well water to a depth of 44 cm and covered each with fiberglass screening (1 mm mesh) to prevent colonization by insects and amphibians. On 17 May, we added 2.5 L of water from three small local ponds (Middleton, Ebaugh, and Olde Minnow) to each mesocosm. On 21 May, we added 1.25 L aliquots of zooplankton collected using 3 1‐m vertical tows of a 153 μm zooplankton net per 3.78 L from Ebaugh Pond. These three ponds differ in the fish community present, as well as their size and productivity, and therefore provide a variety of zooplankton communities (Rettig, Schuman, and McCloskey 2006). By using these three ponds, we hoped to provide a diverse zooplankton community in the mesocosms. We added an additional 1.25 L aliquot of zooplankton collected using 4 1‐m vertical tows of a 153 μm zooplankton net per 3.78 L from Olde Minnow Pond on 24 May. On 27 May, we added artificial vegetation (strands of plastic rope) to each mesocosm at a density that visually approximated the density observed in local ponds. Rope has been used successfully in previous experiments to simulate aquatic vegetation (Savino and Stein 1982). We added a final 1 L aliquot of zooplankton collected using 3 1.5‐m vertical tows of a 153 μm zooplankton net per 3.78 L from Middleton Pond to each mesocosm on 28 May. We did not include leaf litter that is often used in mesocosm experiments to provide nutrients or substrates for zooplankton and algae. However, other mesocosm experiments we have perfomed using similar methods (i.e., no leaf litter) found sufficient zooplankton and algal resources to support the fish densities used in our experiment, even during less productive months (e.g., Geyer, Smith, and Rettig 2016). The lack of the additional resources might have reduced the abundance of zooplankton and algae in the mesocosms and thus would increase the potential for competition; however, fish in similar mesocosm experiments rapidly lowered zooplankton abundances to very low levels even with leaf litter (Rettig and Smith 2021; Rettig, Teeters, and Smith 2021). In addition, it seems unlikely that the lack of leaf litter had a major effect on our results since both species showed positive changes in body size or recruitment, as well as high survivorship, in at least some treatments (see Results), suggesting that fish had access to sufficient zooplankton resources in the experiments.

We randomly assigned each mesocosm to one of the seven treatments (each replicated six times): control (no fish), low density *G. affinis* (4 fish; 2 males, and 2 females), high density *G. affinis* (8 fish; 4 males, and 4 females), low density juvenile *L. macrochirus* (4 fish), high density juvenile *L. macrochirus* (8 fish), low density mixed species (2 *G. affinis* [1 male, 1 female], 2 juvenile *L. macrochirus*), and high density mixed species (4 *G. affinis* [2 males, 2 females], 4 juvenile *L. macrochirus*). The total length ranged from approximately 25–30 mm for male *G. affinis*, 30–40 mm for female *G. affinis*, and 35–65 mm for juvenile *L. macrochirus*. These densities are in the range of densities of these species in local ponds (Rettig and Arrington, n.d.). In addition, previous mesocosm experiments involving *L. macrochirus* or *G. affinis* from these populations found that the densities (or lower) and body sizes used in our experiment resulted in reductions in zooplankton abundances and affected insect and amphibian abundance or colonization, while demonstrating relatively high survivorship (*G. affinis*, Christenson et al. 2014; Geyer, Smith, and Rettig 2016; Smith and Harmon 2019; Harmon and Smith 2021; Rettig and Smith 2021; *L. macrochirus*: Smith et al. 2016; Rettig, Teeters, and Smith 2021), suggesting these are reasonable densities for our experiments. This design allowed us to examine the effects of both intra‐ and interspecific competition. On 4 June, we began the experiment by adding the appropriate number and type of fish to each mesocosm. We collected *L. macrochirus* using a seine from Middleton Pond and captured *G. affinis* using dip nets from Olde Minnow Pond. We recorded the total length (TL) and mass of each *G. affinis* and *L. macrochirus* prior to stocking.

Using tube samplers (Rettig 2003; Aday et al. 2005; Chase et al. 2009), we collected zooplankton samples from three predetermined spots in each mesocosm on five sampling dates: 2 June, 9 June, 16 June, 30 June, and 14 July. Water collected from tube samplers (0.68 L) was filtered through a 63 μm mesh sieve and the filtered zooplankton was pooled for each mesocosm and preserved in acid Lugol's solution. We subsequently used a Nikon SMZ800 dissecting microscope (Nikon Instruments Inc., Melville, New York) to identify and count the zooplankton. We counted all zooplankton in the sample which included cladocerans (*Alona*, *Bosmina*, *Ceriodaphnia*, *Chydorus*, *Scaphaloberus*, and *Simocephalus*), copepods (both cyclopoid and calanoid), rotifers (*Brachionus*, *Keratella*, *Lecane*, *Platyias*), and ostracods. We divided the counts by the volume of water sampled to calculate the number of zooplankton per liter.

On 15 July, we removed all surviving fish from the mesocosms and recorded the number of fish and measured their TL and final mass. At the end of the experiment, we discovered that one of the low density, mixed fish mesocosms had inadvertently been stocked with too many *L. macrochirus* and so was excluded from all analyses. We calculated a survivorship/recruitment index (SRI) for *G. affinis* by dividing the number of individuals (including adults, juveniles, and larvae) present in each mesocosm at the end of the experiment by the initial number of adults introduced into each mesocosm to account for recruitment in the mesocosms (the juvenile *L. macrochirus* did not reproduce in the experiment). Newly recruited *G. affinis* were easy to distinguish from the adults that were stocked at the beginning of the experiment since the recruits were much smaller and had a different appearance compared to the adults (i.e., they were obviously fry). We also calculated a body condition index (BCI) by dividing body mass by TL and mass change by subtracting the mean initial mass in each mesocosm from the mean final mass of *L. macrochirus*.

We used two‐way analyses of variance to examine the effects of competition type (intraspecific vs. interspecific) and fish density (low vs. high) and their interaction on body size or growth, BCI, and survivorship (arcsin square root transformed) or SRI for each fish species. For analyses of count data (e.g., total number of *G*. affinis at the end of the experiment), we used a generalized linear model with a Poisson distribution and log link. We used a one‐way ANOVA to compare the numbers of total zooplankton (cladocerans, copepods, rotifers, ostracods)and large zooplankton (*Scaphaloberus, Daphnia, Ceriodaphnia,* and *Simocephalus*) in the initial sample (2 June) prior to the implementation of treatments. We used a one‐way repeated‐measures analysis of variance to examine the effects of experimental treatments (including the fishless control) on the total number of zooplankton (L^−1^) and the number of large zooplankton (L^−1^). We used JMP Pro 16.0 (SAS Institute, Cary, NC) for the statistical analyses. We used an α‐value of 0.05 for significance. Means are given ±1 S.E.

### 2.2 Experiment 2 (2012)

We filled 48 mesocosms (1136 L capacity, 63.5 cm height, 175 cm diameter) with well water to a depth of 44 cm and covered each with a fiberglass screen (1 mm mesh). We inoculated each tank with a 2 L aliquot of pond water from Olde Minnow Pond on 11 May. We collected zooplankton from Middleton Pond using vertical tows at a depth of 2 m with a 153 μm net and added a 370 mL aliquot (the equivalent of one vertical tow at 2 m depth) of zooplankton and pond water to each mesocosm on each of three consecutive days (22–24 May), as well as on 6 June.

We assigned each mesocosm to one of eight treatment combinations (each replicated six times) based on the manipulation of three variables: the presence or absence of vegetation, the presence or absence of juvenile *L. macrochirus*, and the presence or absence of adult *G. affinis*. On 13 June, we placed plastic aquarium plants and strands of yellow rope into the vegetation present treatments, to simulate vegetation. Artificial plants appear to support similar numbers of macroinvertebrates as natural plants (Gerrish and Bristow 1979), and *G. affinis* do not appear to differentiate between real and artificial plants (Casterlin and Reynolds 1977).

On 14 June, we started the experiment by adding two juvenile *L. macrochirus* (total length = 34–65 mm) to each *L. macrochirus* mesocosm and four adult *G. affinis* to each *G. affinis* mesocosm. Initially, we stocked two male (total length = 24–29 mm) and two female (total length = 31–41 mm) *G. affinis* but due to high male mortality early in the experiment and the limited availability of male *G. affinis*, some of the males were replaced with females. Male mortality may have been higher than females because they are smaller and we have noted that their gonopodium can be easily damaged during handling. We collected juvenile *L. macrochirus* by seining at Middleton Pond and *G. affinis* by dipnetting in Olde Minnow Pond. We recorded the TL and mass of each *L. macrochirus* and the TL of each *G. affinis* prior to stocking into the mesocosms. To allow invertebrate and amphibian colonization throughout the experiment, we did not cover mesocosms. By the end of the experiment, the mesocosms were colonized by Gray Treefrogs (*Hyla versicolor*) and a variety of insects (Corixidae, Dytiscidae, Gerridae, Hydrophilliidae, and Notonectidae).

We collected zooplankton samples from each tank on six sampling dates: 14, 18, 21, 28 June, 9, and 12 July using the same methods as in Experiment 1. We counted and identified zooplankton using the methods used in Experiment 1.

On 13 July, we removed and counted surviving fish from mesocosms and recorded the TL and final mass of each fish. We calculated SRI and BCI for each species as in Experiment 1. We calculated changes in TL or BM for *L. macrochirus* and *G. affinis* by subtracting initial mean TL or BM from the final TL or BM, respectively.

We used two‐way analyses of variance to examine the effects of competitor presence or absence and vegetation treatments and their interaction on each fish species, on change of total length, change of mass, change of BCI, and survivorship (arcsin square root transformed) for *L. macrochirus* and SRI, number of larvae, and change of total length for *G. affinis*. For analyses of count data (e.g., number of larval *G. affinis*), we used a generalized linear model with a Poisson distribution and log link. We used a three‐way ANOVA to compare the initial numbers of total zooplankton (cladocerans, copepods, rotifers, ostracods) and large zooplankton (*Scaphaloberus, Daphnia, Ceriodaphnia,* and *Simocephalus*) on the initial sample (2 June) prior to the implementation of treatments. We used a three‐way repeated‐measures analysis of variance to examine the effects of vegetation, *L. macrochirus*, and *G. affinis* treatments on the total number of zooplankton (L^−1^) and the number of large zooplankton (L^−1^). We used JMP Pro 16.0 (SAS Institute, Cary, NC) for the statistical analyses. We used an α‐value of 0.05 for significance. Means are given ±1 S.E.

## 3 Results

### 3.1 Experiment 1

#### 3.1.1 Lepomis macrochirus

**FIGURE 1:**
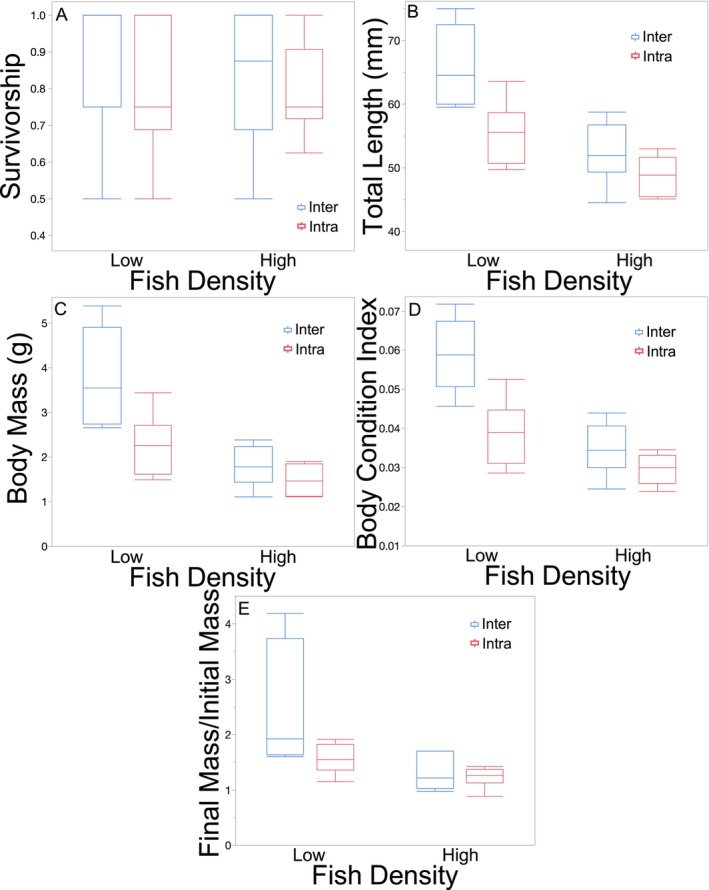
Boxplots with outliers of (A) survivorship, (B) total length, (C) body mass, (D) body condition index, and (E) body mass change of *Lepomis macrochirus* as a function of fish density and the presence (interspecific competition) or absence (intraspecific competition) of *Gambusia affinis* in Experiment 1.

*Lepomis macrochirus* survivorship was not affected by competition type (Figure 1A; Table 1). Fish density had no effect on *L. macrochirus* survivorship (Figure 1A; Table 1). The interaction between competition type and fish density had no effect on the survivorship of *L. macrochirus* (Figure 1A; Table 1).

**TABLE 1:** Results of two‐way analyses of variance examining the effects of competition type (interspecific vs. intraspecific) and fish density (low or high) on the survivorship, total length (TL), mass, body condition index (BCI), and mass change of *Lepomis macrochirus* in Experiment 1.

	df	Survivorship		TL		Mass		BCI		Mass change	
		*F*	*p*	*F*	*p*	*F*	*p*	*F*	*p*	*F*	*p*
Competition type	1	1.89	0.19	11.79	0.0028	9.68	0.0058	16.94	0.0006	4.68	0.043
Density	1	0.51	0.48	24.18	< 0.0001	22.12	0.0002	29.5	< 0.0001	10.31	0.0046
Competition type × Density	1	0.17	0.68	2.85	0.11	4.12	0.057	5.92	0.025	3.42	0.08
Error	19										

Mean *L. macrochirus* TL was 12% greater under interspecific competition than under intraspecific competition (Figure 1B; Table 1). *Lepomis macrochirus* in the high fish density mesocosms had a mean TL that was 10 mm smaller than those in low fish density mesocosms (Figure 1B; Table 1). The interaction between competition type and fish density was not significant (Figure 1B; Table 1).

Mean *L. macrochirus* mass was 44% greater in the interspecific competition treatment than the intraspecific competition treatment (Figure 1C; Table 1). Mean *L. macrochirus* mass in the high fish density mesocosms was almost 50% of the mean in low fish density mesocosms (Figure 1C; Table 1). There was a tendency for fish density to have a greater effect under interspecific competition than intraspecific competition (Figure 1C; Table 1).

Mean BCI of *L. macrochirus* was 35% greater under interspecific competition than intraspecific competition (Figure 1D; Table 1). Mean BCI was greater at low fish density than that at high fish density (Figure 1D; Table 1). The effect of fish density was greater under interspecific competition than intraspecific competition (Figure 1D; competition type*fish density interaction; Table 1).

Mean mass change of *L. macrochirus* was 34% greater under interspecific competition than under intraspecific competition (Figure 1E; Table 1). Mean mass change of *L. macrochirus* was almost 60% greater at low fish density than at high fish density (Figure 1E; Table 1). The interaction between competition type and fish density was not significant, but there was a tendency for the effect of competition type to be stronger at low density than at high density (Figure 1E; Table 1).

#### 3.1.2 Gambusia affinis

**FIGURE 2:**
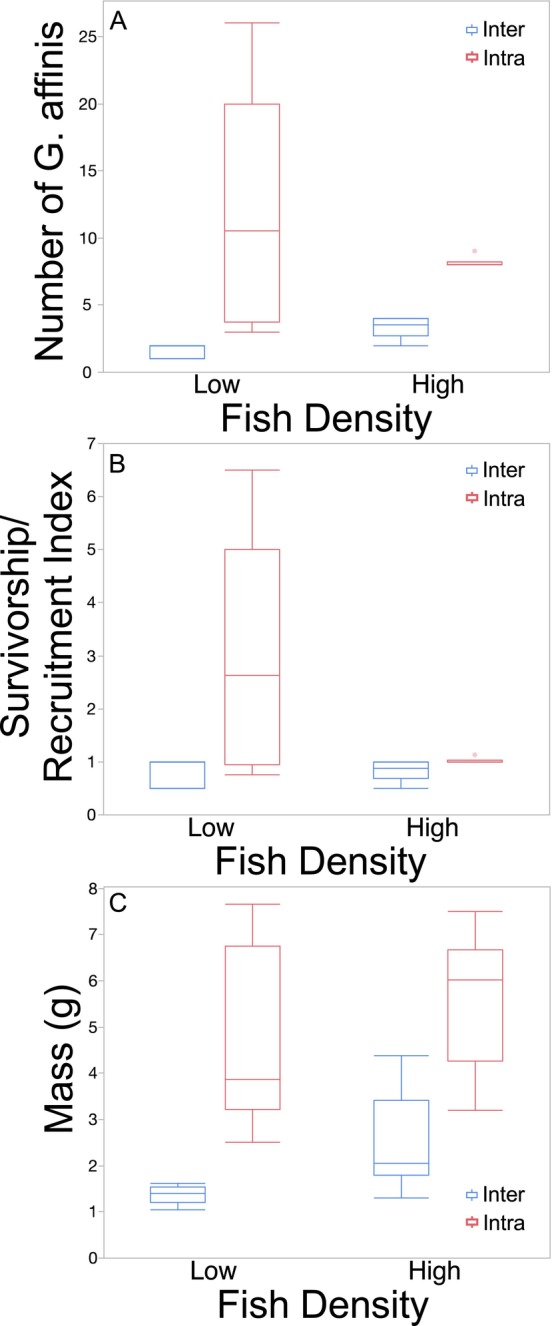
Boxplots with outliers of (A) number at end of experiment, (B) survivorship/recruitment index, and (C) total mass of *Gambusia affinis* as a function of fish density and the presence (interspecific competition) or absence (intraspecific competition) of *Lepomis macrochirus* in Experiment 1.

The total number of *G. affinis* in a mesocosm at the end of the experiment was four times greater in intraspecific competition mesocosms than in interspecific competition mesocosms (Figure 2A; χ^2^_1_ = 55.2, *p* < 0.0001). Fish density did not affect the total number of *G. affinis* in a mesocosm at the end of the experiment (Figure 2A; χ^2^_1_ = 0.60, *p* = 0.44). The interaction of competition type and fish density was significant with the difference between intra‐ and interspecific competition mesocosms greater at low density (Figure 2A; χ^2^_1_ = 6.52, *p* = 0.01).

The mean SRI of *G. affinis* in the intraspecific competition mesocosms was more than two times the mean SRI in the interspecific competition mesocosms (Figure 2B; Table 2). The SRI tended to be higher at low fish density than high fish density, but this was not statistically significant (Figure 2B; Table 2). There was also a nearly significant tendency for the SRI to be low in the interspecific treatments (at both low and high fish density), low in the high fish density intraspecific treatments, and higher in the low density intraspecific treatments (Figure 2B; Table 2).

**TABLE 2:** Results of two‐way analyses of variance examining the effects of competition type (interspecific vs. intraspecific) and fish density (low or high) on survivorship‐recruitment index (SRI) and total mass at the end of the experiment (mass) of *G. affinis* in Experiment 1.

	dfs	SRI		Mass	
		*F*	*p*	*F*	*p*
Competition type	1	5.22	0.034	30.09	< 0.0001
Density	1	3.47	0.078	3.17	0.09
Competition type × Density	1	3.71	0.069	0.0093	0.92
Error	19				

The total mass of *G. affinis* in a mesocosm at the end of the experiment was almost 2.5 times greater in the intraspecific competition mesocosms than in the interspecific competition mesocosms (Figure 2C; Table 2). Total mass of *G. affinis* did not differ between low and high fish density (Figure 2C; Table 2). The interaction of competition type and fish density was not significant (Figure 2C; Table 2).

#### 3.1.3 Zooplankton

For the initial zooplankton samples on 2 June, there were no differences among treatments in the mean total number of zooplankton per liter (*F*_6,34_ = 0.22, *p* = 0.97). The overall mean (±1 S.E.) number of total zooplankton at the start of the experiment was 588.7 ± 47.4 individuals L^−1^. There was also no difference in the mean number of large zooplankton per liter among the treatments (*F*_6,34_ = 0.44, *p* = 0.85). The overall mean (±1 S.E.) number of large zooplankton at the start of the experiment was 104.9 ± 12.8 individuals L^−1^.

**FIGURE 3:**
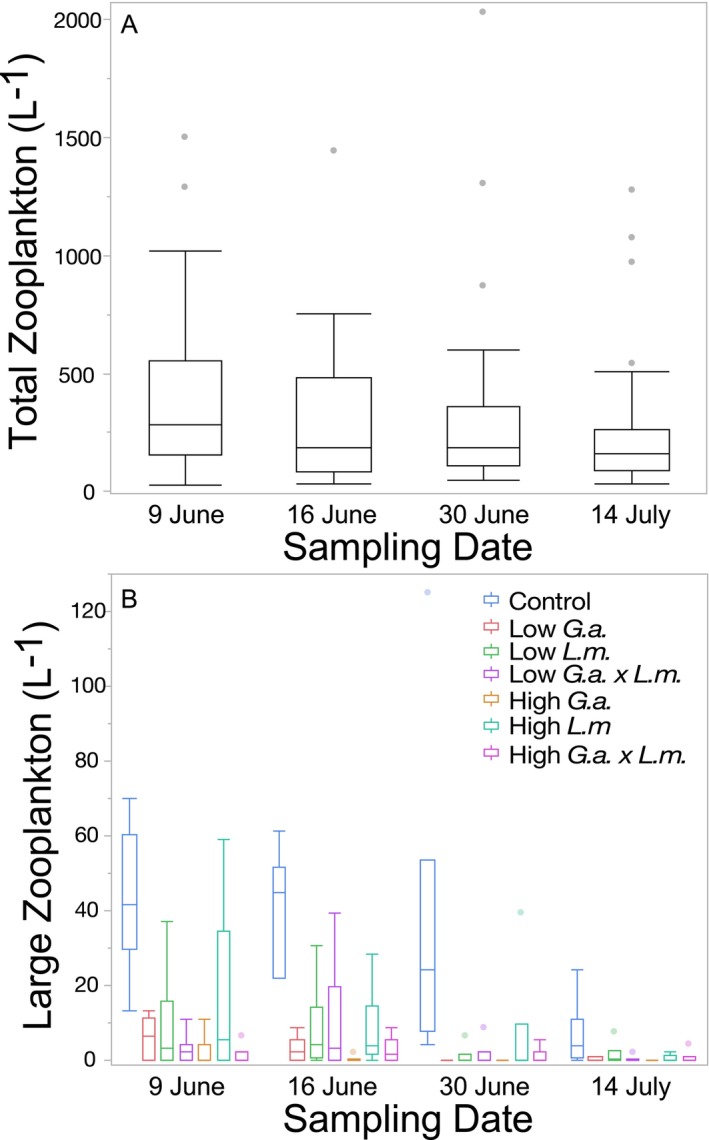
Boxplots with outliers of (A) total number of zooplankton per liter over the course of Experiment 1 and (B) the number of large zooplankton per liter in each treatment over the course of Experiment 1.

Across the experiment, the total abundance of zooplankton did not differ among treatments (Table 3). The total abundance of zooplankton did not change over the course of the experiment (Figure 3A; Table 3). There was no interaction between time and treatment (Table 3). For large zooplankton, the control treatment (i.e., no *L. macrochirus* or *G. affinis*) had significantly more individuals per liter compared to all treatments with fish (Figure 3B; Table 3). The abundance of large zooplankton declined over the course of the experiment (Figure 3B; Table 3). The number of large zooplankton in the control mesocosms was greater for all sampling dates except the last sampling date, 14 July (Figure 3B; sampling date * treatment interaction; Table 3).

**TABLE 3:** Results of repeated measures analyses of variance examining the effects of treatment combinations of fish species composition and density on the total number of zooplankton and the number of large zooplankton (*Scaphaloberis*, *Daphnia, Ceriodaphnia*, and *Simocephalus*) over the course of Experiment 1.

	dfs	Total zooplankton		Large zooplankton	
		*F*	*p*	*F*	*p*
Treatment	6,34	0.52	0.79	11.51	< 0.0001
Time	3102	2.32	0.08	6.72	0.0003
Time × Treatment	18,102	0.79	0.70	1.77	0.040

### 3.2 Experiment 2

#### 3.2.1 Lepomis macrochirus

**FIGURE 4:**
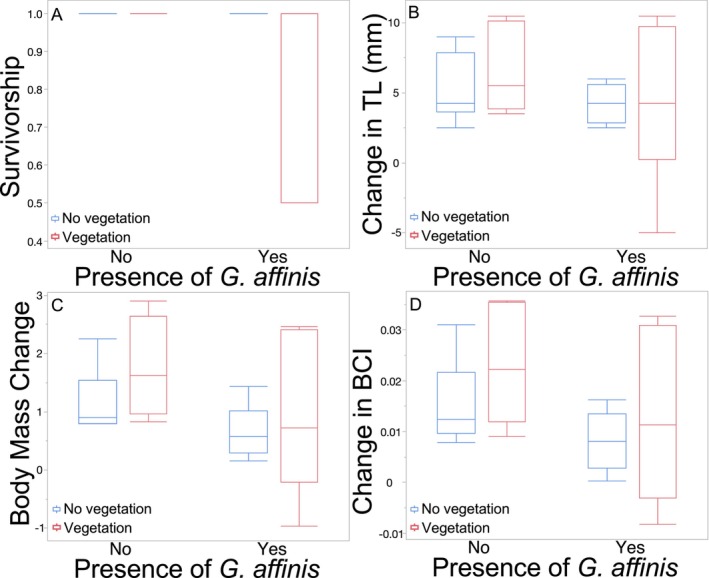
Boxplots with outliers of (A) survivorship, (B) growth in total length, (C) body mass change, and (D) change in body condition index (BCI) of *Lepomis macrochirus* as a function of the presence or absence of *Gambusia affinis* and the presence or absence of vegetation in Experiment 2.

Survivorship of *L. macrochirus* was generally high (overall = 0.96 ± 0.029). Survivorship of *L. macrochirus* was not affected by *G. affinis* treatment (Figure 4A; Table 4), vegetation treatment (Figure 4A; Table 4), or their interaction (Figure 4A; Table 4).

**TABLE 4:** Results of two‐way analyses of variance examining the effects of the presence or absence of *Gambusia affinis* and the presence or absence of vegetation on the survivorship, growth in total length (growth), mass change, and BCI change of *Lepomis macrochirus* in Experiment 2.

	dfs	Survivorship		Growth		Mass change		BCI change	
		*F*	*p*	*F*	*p*	*F*	*p*	*F*	*p*
*G. affinis*	1	2.5	0.13	1.26	0.28	3.58	0.073	3.40	0.08
Vegetation	1	2.5	0.13	0.19	0.67	1.35	0.26	1.60	0.22
*G. affinis* × Vegetation	1	2.5	0.13	0.19	0.67	0.25	0.62	0.097	0.76
Error	20								

Mean TL growth of *L. macrochirus* was not affected by the presence or absence of *G. affinis* (Figure 4B; Table 4). Mean TL growth of *L. macrochirus* was not affected by vegetation (Figure 4B; Table 4). The interaction of vegetation and *G. affinis* presence was not significant (Figure 4B; Table 4).

Mean mass change for *L. macrochirus* did not differ between mesocosms with and without *G. affinis* (Figure 4C; Table 4). Mean mass change for *L. macrochirus* did not differ between mesocosms with and without vegetation (Figure 4C; Table 4). The interaction between vegetation and *G. affinis* presence was not significant (Figure 4C; Table 4).

The mean change in BCI was not affected by the presence or absence of *G. affinis* (Figure 4D; Table 4). The mean change in BCI was not affected by vegetation (Figure 4D; Table 4). The interaction between vegetation and *G. affinis* presence was not significant (Figure 4D; Table 4).

#### 3.2.2 Gambusia affinis

**FIGURE 5:**
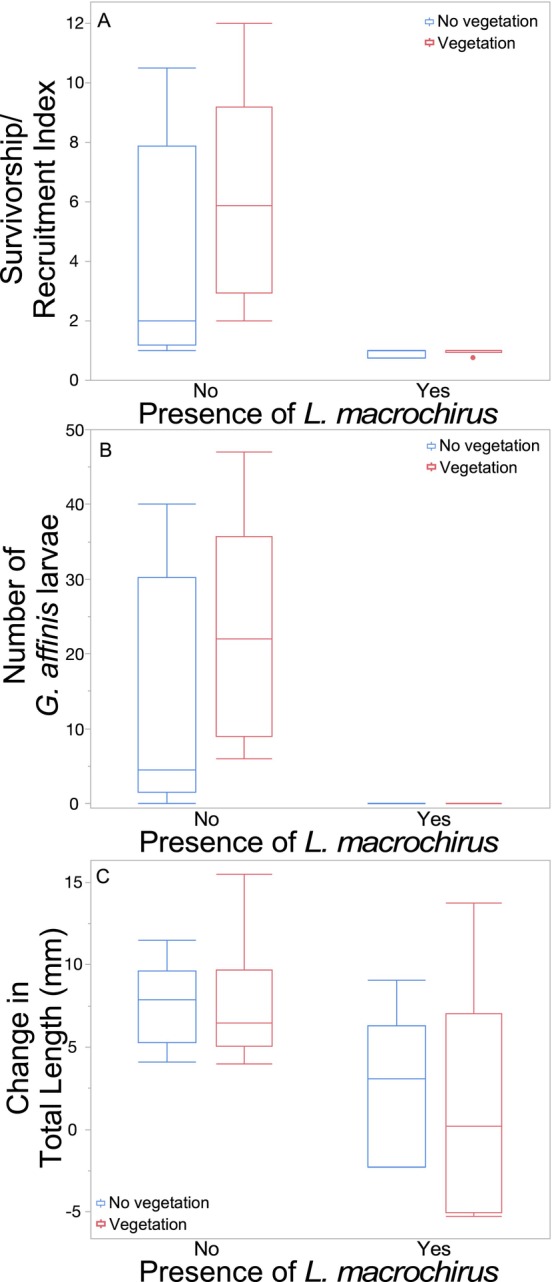
Boxplots with outliers of (A) survivorship/recruitment index, (B) number of larvae, and (C) total length change of *Gambusia affinis* as a function of the presence or absence of *Lepomis macrochirus* and the presence or absence of vegetation in Experiment 2.

The SRI was over five times greater in mesocosms without *L. macrochirus* than in mesocosms with *L. macrochirus* (Figure 5A; Table 5). The SRI of *G. affinis* was not affected by vegetation (Figure 5A; Table 5). The interaction between vegetation and *L. macrochirus* was not significant (Figure 5A; Table 5).

**TABLE 5:** Results of two‐way analyses of variance examining the effects of the presence or absence of *Lepomis macrochirus* and the presence or absence of vegetation on the survivorship‐recruitment index (SRI) and mean total length change (TL change) of *Gambusia affinis* in Experiment 2.

	df	SRI		TL change	
		*F*	*p*	*F*	*p*
*L. macrochirus*	1	13.98	0.0013	7.94	0.01
Vegetation	1	1.07	0.31	0.11	0.74
*L. macrochirus* × Vegetation	1	0.99	0.33	0.09	0.77
Error	20				

There were significantly more *G. affinis* larvae in mesocosms without *L. macrochirus* than those with *L. macrochirus*, indeed no larvae were found in mesocosms with *L. macrochirus* (Figure 5B; χ^2^_1_ = 283.4, *p* < 0.0001). The mean number of *G. affinis* larvae did not differ between mesocosms with and without vegetation (Figure 5B; χ^2^_1_ < 0.001, *p* = 0.99). The interaction between vegetation and *L. macrochirus* presence was not significant (Figure 5B; χ^2^_1_ < 0.001, *p* = 0.99).

Mean TL change in adult *G. affinis* was almost four times greater in mesocosms without *L. macrochirus* than in mesocosms with *L. macrochirus* (Figure 5C; Table 5). However, mean TL change was not affected by vegetation (Figure 5C; Table 5), and the interaction between vegetation and *L. macrochirus* was not significant (Figure 5C; Table 5).

#### 3.2.3 Zooplankton

For the initial zooplankton samples on 14 June, there were no difference in mean total number of zooplankton per liter between the vegetation treatments (*F*_1,40_ = 0.41, *p* = 0.53) or the *G. affinis* treatments (*F*_1,40_ = 0.13, *p* = 0.72). The overall mean (±1 S.E.) number of total zooplankton at the start of the experiment was 209.7 ± 11.4 individuals L^−1^. There were more zooplankton in mesocosms assigned to the *L. macrochirus* present treatment than in mesocosms assigned to the *L. macrochirus* absent treatment (233.5 ± 17.8 [*N* = 24] vs. 185.8 ± 13.0 [*N* = 24]; *F*_1,40_ = 4.24, *p* = 0.046). None of the interaction terms were significant (all *p* > 0.45). On 14 June, there were no differences in the mean number of large zooplankton per liter between the vegetation treatments (*F*_1,40_ = 0.38, *p* = 0.54), the *L. macrochirus* treatments (*F*_1,40_ = 1.78, *p* = 0.19), or the *G. affinis* treatments (*F*_1,40_ = 3.25, *p* = 0.08). No interaction terms were significant (all *p* > 0.32). The overall mean (±1 S.E.) number of large zooplankton at the start of the experiment was 117.9 ± 8.8 individuals L^−1^.

**FIGURE 6:**
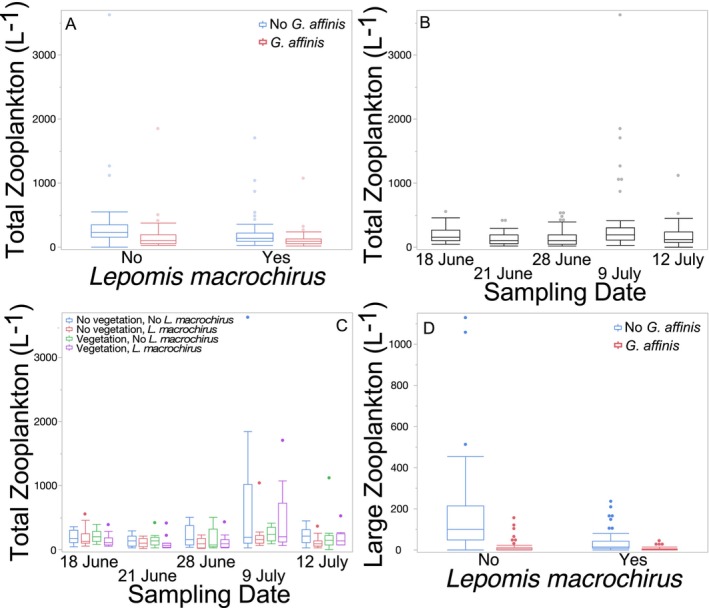
Boxplots with outliers of (A) total number of zooplankton per liter in the presence or absence of *Lepomis macrochirus* and the presence or absence of *Gambusia affinis*, (B) total number of zooplankton per liter over the course of the experiment, (C) total number of zooplankton per liter in the presence or absence of vegetation and the presence or absence of *L. macrochirus* over the course of the experiment, and (D) number of large zooplankton per liter in the presence or absence of *L. macrochirus* and the presence or absence of *G. affinis* in Experiment 2.

The presence of *L. macrochirus* did not affect total zooplankton abundance (Figure 6A; Table 6), whereas the presence of *G. affinis* reduced the total abundance of zooplankton (Figure 6A; Table 6). The vegetation treatments did not affect total zooplankton abundance (Table 6). The interactions of vegetation and *L. macrochirus* (Table 6) and *G. affinis* (Table 6) were not significant. The interaction of *L. macrochirus* and *G. affinis* was also not significant (Table 6). The three‐way interaction between vegetation, *L. macrochirus*, and *G. affinis* was not significant (Table 6). The total abundance of zooplankton varied across the course of the experiment, generally decreasing for the first three sampling dates then peaking during the July 9 sampling date (Figure 6B; Table 6). There was a significant three‐way interaction between time, vegetation, and *L*. macrochirus, with more zooplankton found in mesocosms with *L. macrochirus* when there was vegetation present, but in mesocosms without *L. macrochirus,* there were more zooplankta when vegetation was present; this was only on the 9 July sampling date (Figure 6C; Table 6). No other interactions with time were significant (Table 6).

**TABLE 6:** Results of three‐way repeated measures analyses of variance examining the effects of the presence or absence of *Lepomis macrochirus* (*Lm*), *Gambusia affinis* (*Ga*), and vegetation (Veg) on the number of total zooplankton and large zooplankton (*Scaphaloberis*, *Daphnia*, *Ceriodaphnia*, and *Simocephalus*) over the course of Experiment 2.

	dfs	Total Zooplankton		Large Zooplankton	
		*F*	*p*	*F*	*p*
*L. macrochirus*	1,40	3.04	0.09	11.56	0.0015
*G. affinis*	1,40	12.70	0.001	31.04	< 0.0001
Vegetation	1,40	0.25	0.62	0.07	0.80
*Lm* × Veg	1,40	2.82	0.10	0.32	0.57
*Ga* × Veg	1,40	0.05	0.83	0.02	0.90
*Lm* × *Ga*	1,40	0.47	0.50	10.10	0.0029
*Lm* × *Ga* × Veg	1,40	0.30	0.59	0.12	0.73
Time	4160	6.08	0.0001	0.70	0.59
*Lm* × Time	4160	0.20	0.94	0.64	0.63
*Ga* × Time	4160	0.56	0.69	0.40	0.81
Veg × Time	4160	0.38	0.82	0.57	0.68
*Lm* × Veg × Time	4160	2.76	0.03	0.43	0.79
*Ga* × Veg × Time	4160	0.58	0.68	0.54	0.71
*Lm* × *Ga* × Time	4160	0.19	0.94	0.80	0.52
*Lm* × *Ga* × Veg × Time	4160	0.69	0.60	0.43	0.79

There were fewer large zooplankton in mesocosms with *L. macrochirus* (Figure 6C; Table 6) and with *G. affinis* (Figure 6C; Table 6). There was also a significant interaction between *L. macrochirus* and *G. affinis* treatments, with *G. affinis* generally having a greater effect on zooplankton than *L. macrochirus* (Figure 6D; Table 6). Vegetation had no effect on the number of large zooplankton (Table 6). None of the two‐way or three‐way interactions involving vegetation were significant (Table 6). The abundance of large zooplankton remained relatively constant over the course of the experiment (Table 6), and no interactions with the sampling date were significant (Table 6).

## 4 Discussion

In our experiments, we found evidence for competitive interactions between *L. macrochirus* and *G. affinis*. However, competition did not appear symmetric, with *L. macrochirus* generally more affected by intraspecific competition than interspecific competition and *G. affinis* more affected by interspecific competition than intraspecific competition. Additionally, it appears that predation on *G. affinis* offspring by *L. macrochirus* and *G. affinis* (at high density) can impact recruitment of *G. affinis*. We also found evidence that zooplankton communities were reduced by the fish in the experiment relative to controls, especially the number of large zooplankton taxa, suggesting competition may occur over this potentially limiting resource.

*Gambusia affinis* were more strongly affected by interspecific interactions than intraspecific interactions, with lower growth rates and SRI with significantly less to virtually no recruitment observed in the interspecific mesocosms in both experiments (i.e., those with *L. macrochirus*). Since our SRI includes larvae produced by the end of the experiment, the observed decrease in *G. affinis* SRI indicates a reduction in *G. affinis* recruitment in mesocosms with *L. macrochirus*. Our results are consistent with *L. macrochirus* consuming the offspring of *G. affinis* in the mesocosms. Indeed, *Lepomis* spp. can be predators on *G. affinis* (e.g., Simkins and Belk 2017) as well as small or larval fish (Seaburg and Moyle 1964; Rettig and Mittelbach 2002; Andraso 2005; Carpenter and Mueller 2008), and piscivorous fish (e.g., *Micropterus salmoides*) can eliminate *G. affinis* from small ponds (Swingle 1949). In addition, the presence of a predator reduced reproduction of *G. holbrooki* (Mukherjee et al. 2014). The reduced growth rates of *G. affinis* in the presence of *L. macrochirus* also suggest that *L. macrochirus* competitively affects *G. affinis* likely due to their effects on the common food items, such as zooplankton, or through interference competition. Previous studies suggest that *G. affinis* can be negatively affected by competition with other fishes (Taylor, Trexler, and Loftus 2001; Rehage, Lopez, and Sih 2020). Indeed, in both our experiments, the zooplankton communities were affected by the presence of both *L. macrochirus* and *G. affinis*, with a noticeable effect of these fish on the number of large zooplankton taxa. The effects of juvenile *L. macrochirus* on zooplankton and macroinvertebrates communities in lakes have been shown to competitively affect other species of fish (Aday et al. 2005). *Lepomis macrochirus* can be competitively superior to other native freshwater fishes (Cooper, Wagner, and Krantz 1971), and there is some evidence that *L. macrochirus* can affect the foraging success of *G. affinis* via interference (Clemmer and Rettig 2019).

The greater interspecific impacts compared to intraspecific impacts on *G. affinis*, and the greater intraspecific impacts compared to interspecific impacts on *L. macrochirus* suggest that *L. macrochirus* have the potential to control *G. affinis* populations in ponds. Indeed, in a local pond, we have observed a decline and near extirpation of a population of *G. affinis* following the “invasion” of another native Centrarchid, *L. megalotis* (Rettig et al. 2024). However, in a nearby pond, long‐term coexistence of *G. affinis* and *L. macrochirus* has been observed in populations that are at low densities due to a winterkill event from which the pond and its fish populations have never fully recovered (Rettig et al. 2024). Our experiments and observations thus suggest that the presence of *L. macrochirus* may provide biotic resistance to invasive *G. affinis* in small ponds. In fact, native predatory fish have been shown to be able to control or suppress populations of non‐native fishes (Santos et al. 2009), including *G. affinis* (Howell et al. 2013). Additionally, the resistance of freshwater fish assemblages to invasion by non‐native fish species may increase with the presence of native fish that consume small or larval non‐native fish (Baltz and Moyle 1993), especially if they are intraguild predators (e.g., Tuckett et al. 2021; Deacon, Fraser, and Farrell 2023). The intraguild predator, *Anablepsoides hartii*, reduced the ability of another fish, *Poecilia reticulata*, to colonize, especially at low propagule sizes (Deacon, Fraser, and Farrell 2023).

It appears that *L. macrochirus,* and other *Lepomis*, likely serve as intraguild predators on *G. affinis* in our experiments. While we do not have any direct evidence of predation of *L. macrochirus* on *G. affinis* in local ponds or in our experiment, the circumstantial evidence is strong (e.g., the absence of larval *G. affinis* in mesocosms with *L. macrochirus*). In addition, several studies have demonstrated a similar effect of *L. macrochirus*, or other *Lepomis*, on other species of fish. *L. macrochirus* consumed the eggs and larvae of invasive *Cyprinus carpio* and significantly reduced their recruitment (Bajer et al. 2012; Silbernagel and Sorensen 2013; Poole and Bajer 2019). Green Sunfish (*L. cyanellus*) and Longear Sunfish (*L. megalotis*) consumed Red Shiner (*Cyprinella lutrensis*) and Bigeye Shiners (*Notropis boops*) and prevented the establishment of populations of *C. lutrensis* (Marsh‐Matthews, Mattews, and Franssen 2011; Marsh‐Matthews et al. 2013). *Lepomis gulosus* preyed upon juvenile and adult *Heterandria formosa* and appeared to reduce populations of *H. formosa* in nature (Richardson, Gunzburger, and Travis 2006).

We also observed some evidence that *G. affinis* may limit their own populations through cannibalism, at least at high density (and in mesocosms). Adult *G. affinis* do eat fish hatchlings (Swingle 1949) and can prey upon conspecific fry (Miura, Takahashi, and Stewart 1979; Lee, Simon, and Perry 2018; Rettig et al. 2018, 2023). However, the high incidence of cannibalism in *G. affinis* in mesocosm experiments may be due to the greater ease of capture in confined spaces in addition to increasing with density (Riesch et al. 2022).

In our experiments, *L. macrochirus* experienced competitive effects on growth, body size, and body condition, with intraspecific competition appearing stronger than interspecific competition in Experiment 1, and *G. affinis* had no effects on *L. macrochirus* in Experiment 2. In neither experiment did competition affect the survivorship of the juvenile *L. macrochirus*. It appears likely that these effects arose through the impacts of fish treatments on the zooplankton abundance and composition that we observed. Indeed, growth of *L. macrochirus* appears to be limited by zooplankton availability (Osenberg et al. 1988), and *L. macrochirus* experience reduced growth under conditions of high fish densities (Osenberg et al. 1988; Shoup et al. 2007).

Although predation on juvenile non‐native fishes by a native predatory fish can be reduced by habitat complexity (Santos et al. 2009), in Experiment 2, we found no effects of vegetation on either *L. macrochirus* or *G. affinis*, and no significant interactions between vegetation and density or competition type. Our results are consistent with previous studies which suggest that habitat complexity or vegetation does not always influence the impacts of competition or predation involving *G. affinis*. For example, the presence of refuge habitats did not influence the predation of *L. cyanellus* on *G. affinis* (Simkins and Belk 2017). The presence of refuge habitats almost doubled the survival of *Fundulus julisia* exposed to *G. affinis* in experimental conditions, but refuge habitat in the field did not increase the abundance of *F. julisia* in the presence of *G. affinis* (Westhoff, Watts, and Mattingly 2013). These results contrast with other studies that have found that the presence of habitat complexity reduces the effects of predatory fishes (e.g., Santos et al. 2009; Alexander et al. 2015). However, the influence of aquatic habitat complexity on intraguild predation relationships of fish can depend on the size of the fish involved, with larger predators being less successful at invading complex environments and smaller predators more successful at invading complex environments (Reichstein et al. 2013). Given that we were studying *G. affinis*, a relatively small fish, and juvenile *L. macrochirus*, it may be that the size of the fish contributed to the absence of an effect of vegetation in our experiment. Indeed, our results may have been different if adult *L. macrochirus* were used. However, juvenile *L. macrochirus* are found more often in areas of lake that had emergent vegetation than in open water areas (Stahr and Kaemingk 2017), so our use of juveniles in the experiment reflects natural distributions.

## 5 Conclusions

Our experiments found that juvenile *L*. macrochirus and *G. affinis* can compete with each other, with *G. affinis* being more affected by interspecific competition with *L. macrochirus* and *L. macrochirus* being more affected by intraspecific competition, presumably mediated by observed changes to the zooplankton community in the presence of fish. Thus, the competitive interaction is asymmetrical. In addition, it appears that juvenile *L*. macrochirus can prevent successful recruitment of *G. affinis* offspring into a population. The consistency of our results across both experiments, with their slightly different designs and their occurrence in different years, gives weight to these conclusions, especially given that Experiment 2 potentially had additional resources available in the form of colonizing macroinvertebrates and amphibians. The most important result of our experiments from a conservation standpoint is that *L. macrochirus* can potentially resist the invasion of invasive *G. affinis* through both competition and predation (i.e., acting as an intraguild predator). Through their negative effects on invasive *G. affinis* via consumption of their young and competition with adults, native *L. macrochirus*, and potentially other native *Lepomis*, appear to be able to reduce the likelihood of successful establishment of *G. affinis* populations (see Marsh‐Matthews, Mattews, and Franssen 2011; Silbernagel and Sorensen 2013; Poole and Bajer 2019; Schofield et al. 2021 for similar examples involving other species of fish). These effects will likely benefit the native aquatic communties of ponds with fish by reducing the densities of *G. affinis* and thereby limiting their negative effects on native fauna, especially small‐bodied native fish.

## Author Contributions

**Jessica E. Rettig:** conceptualization (lead), formal analysis (supporting), investigation (equal), writing – review and editing (equal). **Elizabeth P. Tristano:** conceptualization (supporting), formal analysis (supporting), investigation (equal), writing – review and editing (equal). **Anthony C. Burger:** conceptualization (supporting), formal analysis (supporting), investigation (equal), writing – review and editing (equal). **Geoffrey R. Smith:** conceptualization (lead), formal analysis (lead), investigation (equal), writing – original draft (lead).

## Conflicts of Interest

The authors declare no conflicts of interest.

## Data Availability

All data used in the preparation of this paper are provided in Dryad (https://doi.org/10.5061/dryad.9w0vt4bq8). [https://datadryad.org/stash/share/yq1Oqr7V8Fq7ohqwTlTxIV4ebvGNN1ylzDK8KZ1hWXo].
